# Induced Prion Protein Controls Immune-Activated Retroviruses in the Mouse Spleen

**DOI:** 10.1371/journal.pone.0001158

**Published:** 2007-11-07

**Authors:** Marius Lötscher, Mike Recher, Karl S. Lang, Alexander Navarini, Lukas Hunziker, Roger Santimaria, Markus Glatzel, Petra Schwarz, Jürg Böni, Rolf M. Zinkernagel

**Affiliations:** 1 Institute of Experimental Immunology, University of Zürich, Zürich, Switzerland; 2 Institute of Neuropathology, University of Zürich, Zürich, Switzerland; 3 Swiss National Center for Retroviruses, University of Zürich, Zürich, Switzerland; New York University School of Medicine, United States of America

## Abstract

The prion protein (PrP) is crucially involved in transmissible spongiform encephalopathies (TSE), but neither its exact role in disease nor its physiological function are known. Here we show for mice, using histological, immunochemical and PCR-based methods, that stimulation of innate resistance was followed by appearance of numerous endogenous retroviruses and ensuing PrP up-regulation in germinal centers of the spleen. Subsequently, the activated retroviruses disappeared in a PrP-dependent manner. Our results reveal the regular involvement of endogenous retroviruses in murine immune responses and provide evidence for an essential function of PrP in the control of the retroviral activity. The interaction between PrP and ubiquitous endogenous retroviruses may allow new interpretations of TSE pathophysiology and explain the evolutionary conservation of PrP.

## Introduction

The prion protein (PrP) is essential for the susceptibility to transmissible spongiform encephalopathies (TSE) such as Creutzfeldt-Jacob disease (CJD) in humans, bovine spongiform encephalopathy (BSE) in cattle, scrapie in sheep and goats, and chronic wasting disease (CWD) in deer and elk [1]. In the absence of PrP, TSE symptoms do not develop [2], and the accumulation of misfolded PrP in diseased tissue is a hallmark of TSE. According to the widely accepted protein-only hypothesis, misfolded PrP itself constitutes the infectious agent [3]. Yet, this dogma awaits to be proven and is challenged by strong evidence for the involvement of non-PrP molecules as “partners in crime” [4]. A recent study demonstrated that synthetic poly(A) RNA, added to purified native hamster PrP, is a sufficient stoichiometric cofactor for *in vitro* amplification of misfolded PrP [5].

Remarkably, hamsters inoculated with samples obtained by these amplification experiments developed TSE. Rather unexpectedly though, the disease occurred not only with samples from amplification that had been initiated by a seed of misfolded PrP originating from diseased hamster brain, but likewise with samples from amplification that had started spontaneously, i.e. in the absence of an initial TSE seed. Moreover, strain characteristics of TSE caused with inoculum from seeded and spontaneous *in vitro* amplification were undistinguishable, but they differed from TSE caused with inoculum of misfolded PrP that was used to seed amplification. Hence, TSE strain characteristics of the inoculum generated by *in vitro* amplification seemed to be imprinted not by a seed of misfolded PrP, as the protein-only hypothesis would entail [3], but rather by the accessory poly(A) RNA. The putative role of RNA in TSE is also supported by a related study reported earlier [6], where the accessory component extractable from tissue homogenate and sufficient for *in vitro* amplification of misfolded PrP was recognized to be particular RNA. By other researchers, RNA aptamers specifically interacting with native or misfolded PrP were isolated by in vitro selection [7], [8]. This indicates that in complex mixtures, RNA species may compete for binding to PrP conformers, which could be the basis for TSE strain formation and interferences [9], [10]. However, despite the evidence gained by means of *in vitro* experiments for a cofactor role of RNA in TSE, no TSE-specific RNA has ever been identified. Therefore, the roles of PrP and possible cofactors in TSE have remained controversial.

The physiological function of PrP might be a key towards better understanding TSE pathophysiology. Numerous reports from the past two decades proposed various physiological roles of PrP in processes as diverse as copper homeostasis, leukocyte differentiation or neuritogenesis, indicating that PrP may serve more than one purpose [4], [11]. In addition, PrP is a broadly expressed and highly conserved gene product [12], [13], suggesting that susceptibility to TSE, which could be considered a negative selection criterion for this protein, is outweighed by a vital function of PrP. Though, this view is in harsh contrast with observations that PrP seems to be dispensable for normal development and health of individual mammals [14], [15]. Up to date, no concept has emerged that would reconcile the conflicting aspects of this enigmatic protein.

We have recently described an up-regulation of PrP in the germinal centers of mouse spleens following immune-stimulation [16]. Although this regulation is suggestive of an immunological role of PrP, no consequences of the increased PrP level could be demonstrated. Here we report that PrP up-regulation occurs in response to massive appearance of endogenous murine retroviruses in the germinal centers upon immune-stimulation. We show that elevated PrP expression helps to reduce the retroviruses to the level before their activation, whereas in the absence of PrP virus abundance persists. Hence, we have identified in the mouse a key role of PrP in the control of activated endogenous retroviruses. Our findings may resolve the aforementioned conflict in the conception of PrP function and suggest a plausible candidate for an RNA species possibly involved in TSE.

## Results

### Co-localized massive appearance of endogenous murine retroviruses and increased PrP expression in immune-stimulated splenic FDC networks

**Figure 1:**
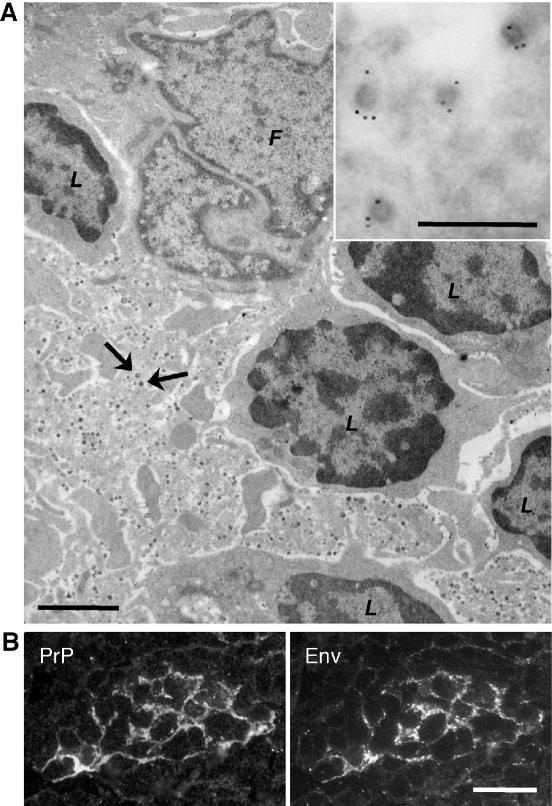
Microscopic revelation of numerous MLV particles in FDC network with increased PrP expression after immune-stimulation of a C57Bl/6 mouse with IC. (A) Ultrastructure of splenic FDC revealed by TEM. Large panel displays plastic section 70 nm thin, showing lymphocytes with dark round nuclei (L), and part of an FDC with lobate nucleus (F) and labyrinthine extensions. Small dark spots (arrows) on FDC extensions represent viral particles. Bar, 2.5 µm. Inset shows cryosection 90 nm thin, depicting viral particles on FDC extension. Dot-like gold particles represent immunostaining specific for MLV Env protein. Bar, 0.5 µm. (B) Co-localized immunofluorescence staining of PrP and MLV Env in splenic germinal center. Semithin (400 nm) cryosection was double-stained with rabbit serum specific for PrP and rat monoclonal antibody specific for MLV Env. Bar, 25 µm.

Splenic PrP up-regulation was observed 8 days after infection of naïve mice with vesicular stomatitis virus (VSV) or intravenous administration of preformed immune complexes [16]. By immunofluorescence staining, up-regulated PrP was detected in the network of follicular dendritic cells (FDC), but its exact localization with respect to FDC dendrites and adjacent lymphocytes remained unknown. Therefore, we examined splenic germinal centers of immune-stimulated mice by transmission electron microscopy (TEM). Staining of ultrathin sections with PrP specific antibodies yielded an immunogold labeling confined to FDC dendrites (Figure S1). Surprisingly, dendrites of immune-stimulated and PrP-expressing FDC were decorated with numerous electron-dense, enveloped particles resembling type C retroviruses (Figure 1A). Their retroviral nature was assessed by staining with a rat antibody specific for the envelope protein (Env) of murine leukemia retroviruses (MLV), resulting in an immunogold labeling on the outer face of the virus particles (Figure 1A inset). In contrast to Env, no PrP presence was revealed in the viral particles, despite morphological evidence for MLV budding from the PrP-expressing FDC (Figure S1).

In fluorescence microscopy, three different antibodies specific for MLV envelope glycoprotein and capsid protein p30, respectively, labeled structures on spleen cryosections that co-localized to FDC networks with strong PrP immunostaining (Figure S2). Whereas MLV staining appeared granular, PrP staining seemed continuous along the FDC dendrites (Figure 1B).

**Figure 2:**
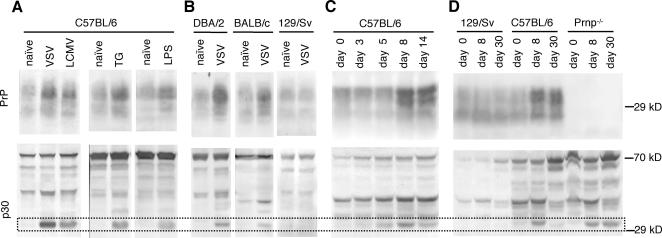
Immunoblots showing increase of PrP and mature MLV p30 in spleens of immune-stimulated mice. (A) Comparison of PrP and mature p30 abundance following various immune-stimuli. C57Bl/6 mice were infected with VSV or LCMV, or treated with thioglycolate (TG) or lipopolysaccharide (LPS). Eight days after immune-stimulation spleen homogenates were analyzed by Western blot for PrP (upper panels) and p30 (lower panels). Representative blots are shown. Protein bands revealing mature p30 protein are boxed. (B) Capacity of various inbred mouse strains to respond to VSV infection with increased abundance of PrP and p30. Mice of the strains DBA/2, BALB/c and 129/Sv were infected with VSV. Splenic PrP and MLV p30 were analysed on day 8 after infection. Representative blots are shown. (C) Kinetic of PrP and p30 abundance following VSV infection. Spleens of C57BL/6 mice were analyzed on day 0, 3, 5, 8 and 14, respectively, after infection. In the presented Western blots homogenates of three spleens per time point group were pooled for analysis, in order to obtain averaged results. (D) Different kinetics of PrP and p30 abundance in 129/Sv, C57BL/6 and Prnp^−/−^ (Nagasaki) mice. Spleens were analyzed on day 0, 8 and 30, respectively, after infection. Per experimental group homogenates of three spleens were pooled.

We used the p30-specific antibody for Western blot analysis of spleen homogenates from naïve and VSV-infected C57BL/6 mice to assess whether the observed MLV particles were associated with the formation of mature p30 protein. In addition to the characteristic band at 30 kDa observed after immune-stimulation, the anti-p30 staining revealed precursor forms of p30 in all tested C57BL/6 spleen samples (Figure 2A). To assure that the 30 kDa band of p30 protein was representative for mature MLV, the activity of reverse transcriptase (RT) in the spleen homogenates was analyzed. The RT activities, as detected by product-enhanced RT (PERT) assay, strictly correlated with the respective p30 signals at 30 kDa (Figure S3). This validated p30-specific immunoblot analysis as a tool to estimate the abundance of matured MLV in splenic tissue.

**Figure 3:**
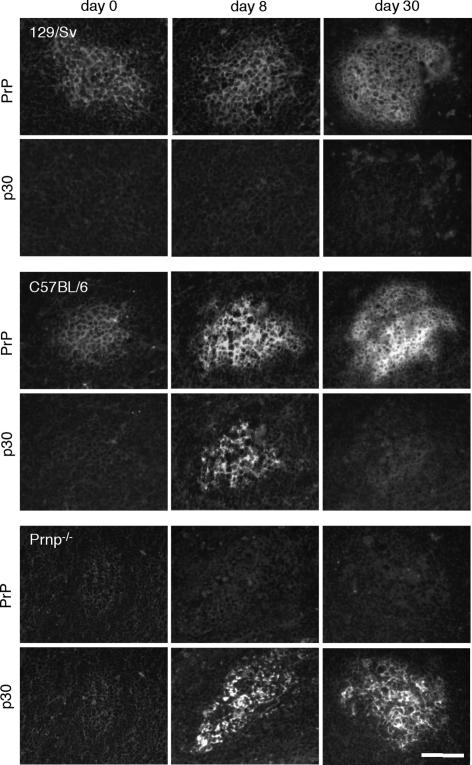
Immunohistochemistry illustrating the interdependence of PrP and IMERV in the splenic FDC network. Frozen spleen sections representing 129/Sv wild-type, C57Bl/6 wild-type and Prnp^−/−^ (Nagasaki) mice on C57Bl/6 background, infected with VSV for 0, 8 and 30 days. Sections were double-stained with rabbit serum specific for PrP (upper row) and goat serum specific for MLV p30 (lower row). Bar, 100 µm.

**Figure 4:**
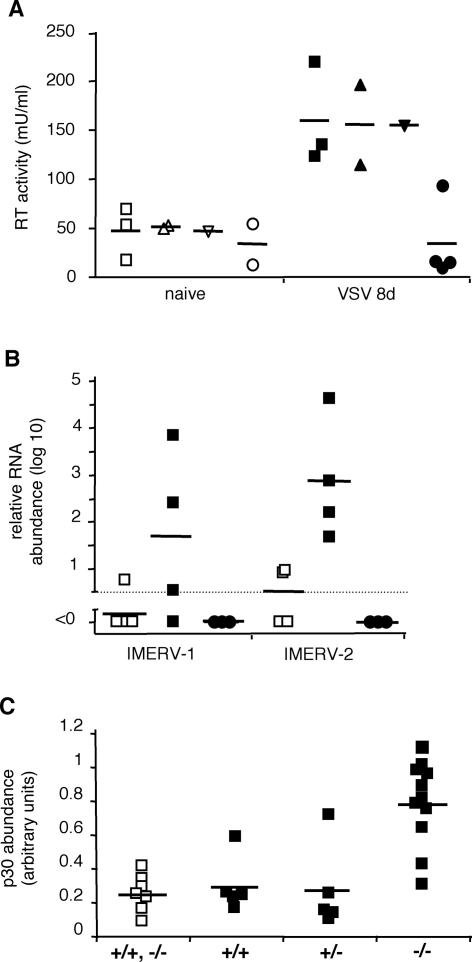
Quantification of splenic IMERV activity. Open plot symbols represent naïve, filled symbols VSV-infected C57Bl/6 (□,▪), BALB/c (▵,▴), DBA/2 (▿,▾) or 129/Sv (○,•) mice. Short horizontal lines indicate average values of respective experimental categories. (A) RT activity in spleen homogenates on day 8 after VSV infection, as revealed by PERT assay. (B) Relative abundance of RNA with IMERV-1 and IMERV-2 specific sequences on day 8 after VSV infection, as assessed by real-time RT-PCR on total splenic RNA. (C) Relative abundance of mature MLV p30 in spleen homogenates on day 30 after VSV infection, as determined by densitometric quantification of immunoblots. Samples were obtained from C57Bl/6 wild-type mice (+/+), Prnp^−/−^ (Nagasaki) mice (−/−) and F1 offsprings of a crossing of the former two lines (+/−).

Additional Western blot analyses revealed that infection of C57BL/6 mice with lymphocytic choriomeningitis virus (LCMV) and administration of thioglycolate or lipopolysaccharide (LPS) also activated endogenous retroviruses (Figure 2A). The broad spectrum of effective immune-stimuli suggested that PrP up-regulation and MLV appearance were consequences of activated adaptive and innate mechanisms of resistance. This is consistent with the fact that splenic PrP up-regulation is dependent on the complement component C1q [16]. Similar to C57BL/6 mice, DBA/2 and BALB/c mice showed augmented MLV activity following immune-stimulation by VSV infection as assessed by p30 Western blot analysis and PERT assay (Figures 2B and 4A), and responded with PrP up-regulation (Figure 2B). In contrast, 129/Sv did not show increased retrovirus activity following VSV immune-stimulation and also failed to up-regulate PrP expression (Figure 2B, 2D, 3, and 4A).

### Partial sequence identification of immune-stimulated MLV

Since the most likely origin of the observed MLV particles is the activation of proviral genes, 129/Sv might lack these particular proviruses, in analogy to the reported paucity of AKR-like proviruses in mouse 129 strains [17]. Clarification of this issue was expected from the identification of MLV-specific proviral genes involved in the observed appearance of MLV particles in immune-stimulated mouse spleens. First, we analyzed total splenic RNA using real-time RT-PCR with a primer-probe set of broad specificity for MLV-related RNA sequences. All spleen samples tested positive for MLV RNA, without significant differences between naïve and immune-stimulated mice (Table 1). This result most likely reflected the general abundance of MLV-related RNA species in mouse spleens, as indicated already by the notable RT “background” activity level of naïve spleen lysates (Figure 4A). Therefore, we isolated MLV particles from immune-stimulated spleen tissue by Env-specific immunoadsorption and subsequently extracted the particle-associated MLV RNA (Figure S4). Complementary DNA corresponding to the purified MLV RNA was amplified and subcloned. From the subcloned PCR products two sequences of 732 nucleotides each were identified. They are homologous to published MLV-related sequences in the NCBI GenBank, with accession number U63133 (nucleotide range 2058–2789) and X94150 (range 2061–2792), respectively. Because these sequences seemed to represent **i**mmune-activated **m**urine **e**ndogenous **r**etro**v**iruses, we named them IMERV-1 (homologous to U63133) and IMERV-2 (X94150). Their up-regulation upon immune-stimulation was confirmed by real-time RT-PCR analysis of total RNA isolated from C57Bl/6 spleens (Figure 4B). Interestingly, no RNA related to IMERV-1 or IMERV-2 was detected in naïve or immune-stimulated 129/Sv spleens (Figure 4B). Genomic IMERV-1 and IMERV-2 sequences were detected in C57Bl/6, DBA/2 and BALB/c mice, but were absent in 129/Sv mice (Table 1). These results corroborated the special status of 129/Sv mice with regard to their retroviral repertoire.

**Table 1:** Quantification of PrP-related RNA, MLV-related RNA (broad specificity), and IMERV-related DNA by real-time (RT-)PCR.

Type of quantified nucleic acid	Sampled mouse cells or spleen tissue	Relative abundance
PrP RNA	3T3 cells, non-infected	100%
	3T3 cells, MLV-infected	93%
MLV RNA (broad)	3T3 cells, non-infected	100%
	3T3 cells, MLV-infected	11'143%
	N2a cells, non-infected	606%
	C57BL/6, naïve	1'300%
	C57BL/6, VSV-infected (8d)	1'213%
	129/Sv, naïve	985%
	129/Sv, VSV-infected (8d)	1'056%
IMERV-1 DNA	C57BL/6	100%
	DBA/2	115%
	BALB/c	123%
	129/Sv	0%
	*Prnp^0/0^* (Zürich)	0%
	F1 (*Prnp^0/0^* (Zürich)×C57BL/6)	50%
	*Prnp^−/−^* (Nagasaki)	100%
IMERV-2 DNA	C57BL/6	100%
	DBA/2	115%
	BALB/c	100%
	129/Sv	0%
	*Prnp^0/0^* (Zürich)	0%
	F1 (*Prnp^0/0^* (Zürich)×C57BL/6)	47%
	*Prnp^−/−^* (Nagasaki)	107%

Comparison of samples was made within sections defined by type of quantified nucleic acid. For the top sample of each section RNA or DNA abundance was arbitrarily set to 100%.

### PrP up-regulation in response to high MLV activity

The IMERV deficiency and the missing splenic PrP up-regulation of 129/Sv mice pointed to a possible dependence of PrP up-regulation on MLV activity. We first tested this hypothesis in a cell culture model of 3T3 fibroblasts infected *in vitro* with MLV. Western blot analysis of cell lysates showed that MLV-propagating cells expressed significantly more PrP than non-infected control 3T3 fibroblasts (Figure S5). Thus, the presence or activity of MLV is associated with increased PrP expression. Importantly, this PrP increase was not correlated with an increase of PrP-related RNA (Table 1), suggesting that a mechanism independent of transcription was involved. Correspondingly, in mice splenic PrP was up-regulated posttranscriptionally after immune-stimulation [16]. This indicated that PrP up-regulation occurred more specifically than just by broad gene activation in response to viral burden.

We examined splenic PrP expression and the presence of MLV p30 over 14 days following immune-stimulation of C57Bl/6 mice by VSV infection (Figure 2C). Retroviral p30 increased at day 5 post-infection, peaked around day 8 and decreased again by day 14. Increased PrP expression was detected by day 8 and persisted beyond day 14. Thus, the kinetics was in line with the assumption that splenic PrP up-regulation occurred in response to IMERV, but also that increased PrP expression might be linked to IMERV regression.

### Retrovirus regression in response to increased PrP expression

If there was an inhibitory effect of up-regulated PrP on IMERV, then PrP-deficient mice should show sustained IMERV activity. To address this subject, *Prnp* knockout mice were assessed for the presence of IMERV and their clearance kinetics.

One line of *Prnp^0/0^* mice [14], which were generated on a mixed 129/Sv-C57BL/6 background, did not show IMERV activity (data not shown) or proviral DNA (Table 1) and was therefore not further evaluated. In contrast, *Prnp* ^−/−^ Nagasaki mice were established on a pure C57BL/6 background [18] and found to be IMERV-competent. They were compared with C57Bl/6 wild-type mice (PrP- and IMERV-competent) and 129/Sv wild-type mice (PrP-competent, IMERV-deficient) on day 8 and day 30 after VSV infection (Figure 2D and 3). In 129/Sv spleens, no MLV p30 was detected at any time point and PrP expression remained low. C57BL/6 wild-type spleens revealed high MLV p30 and PrP presence on day 8, yet on day 30 the two proteins differed. While PrP expression was still high, MLV p30 expression had nearly vanished. In contrast, in C57BL/6 mice lacking PrP (*Prnp^−/−^* Nagasaki) MLV p30 was found enhanced on day 8 and stayed high at least until day 30. Hence, these results support our hypothesis that PrP up-regulation negatively controls the splenic presence of IMERV. An uncertainty about the correct interpretation, however, was caused by the known particularity of *Prnp^−/−^* Nagasaki mice to ectopically express Doppel protein (Dpl), a PrP paralogue, in the brain [19]. Conceivably, Dpl may as well be ectopically expressed in the splenic FDC network, and if so, the persistence of IMERV in *Prnp^−/−^* Nagasaki mice might not be the result of PrP absence but of Dpl presence. Immunohistochemistry indeed revealed that Dpl was expressed in the splenic FDC network of *Prnp^−/−^* Nagasaki mice. In the *Prnp^+/−^* F1 generation of a crossing of C57Bl/6 wild-type and *Prnp^−/−^* Nagasaki mice, both PrP and Dpl were localized in the FDC network (Figure S6). This co-localization permitted us to show that PrP expression in *Prnp^+/−^* F1 mice restored the phenotype of IMERV regression (Figure 4C) irrespective of a putative role of splenic Dpl in IMERV activity.

## Discussion

The present study has revealed that PrP up-regulation occurring in the mouse spleen after immune-stimulation coincides and co-localizes with the transient appearance of endogenous MLV (IMERV). Because PrP up-regulation did not occur in mice that lack the respective IMERV, and in turn, IMERV persisted in the spleen of mice without PrP expression, we conclude that splenic PrP expression plays a crucial role in the negative feedback control of immune-activated endogenous MLV.

Whether PrP expression and retroviral activity are similarly interconnected in non-splenic tissues and other mammalian species remains to be determined, yet, support for such an assumption is provided by cell culture experiments. Over-expression of PrP counteracted the formation of endogenous MLV in N2a neuroblastoma cells (data not shown) and repressed HIV-1 in an infected human cell line [20]. These findings also indicate that the antiretroviral function of PrP might rely on cell-autonomous mechanisms. In the case of HIV-1 repression PrP was reported to interfere with the synthesis of distinct viral proteins on the level of translation [20], implying an interaction with retroviral RNA. In support of such an interaction, functional binding of PrP to retroviral RNA was demonstrated in a cell-free system [21], and PrP was found to be present not only in cell membranes but also in the cytosol [22]. Although PrP binding to retroviral RNA in mammalian cells waits to be confirmed, it is consistent with the concept of PrP and RNA partnering in TSE as summarised in the Introduction. We propose that PrP binding to retrovirus-related RNA is crucial for the antiretroviral effect of PrP expression as well as the pathophysiology of TSE. The latter may explain the interactions observed between TSE and (endogenous) retroviruses in murine *in vitro* and *in vivo* models [23]–[25]. The involvement of retrovirus-related RNA in TSE might also account for the activation pattern of microglia that was found to be reminiscent of an inflammatory response to latent or persistent viruses rather than to amyloidogenic proteins (including abnormal PrP) [26]. Besides the combined evidence for an involvement of retrovirus-related RNA in TSE, it is worth mentioning that in mammals this RNA species occurs ubiquitously, on occasion abundantly, and in various sizes (down to the range of small interfering RNA). Omnipresence or small size could be reasons why no TSE-specific RNA was identified so far. In addition, RNA species can adopt various conformations, and therefore, tissue preparations containing (enriched) TSE infectivity may fail to show TSE-specific ribonucleotide sequences because TSE specificity is imprinted in the secondary structure. According to our model, in which interacting PrP and RNA both can assume TSE-related conformations, principally either binding partner might be able to initiate *per se* propagation of the TSE agent. For the maintenance of propagation and strain characteristics, however, we assume the interaction of both components, PrP and RNA, to be required (in accordance with the results of Deleault et al. [5]).

The massive appearance of IMERV in the FDC network of the mouse spleen is a remarkable finding by itself. Because IMERV activity occurred in response to various immune-stimuli and in several inbred mouse strains, it may be a common trait with physiological functions. Evidence for a connection between endogenous C-type viruses and the humoral immune response of mice was reported already three decades ago [27]. Since then, diverse immunological roles have been discussed for endogenous retroviruses [28] and we may speculate that IMERV activity likewise can provide antiviral resistance or modulate immune responses. The absence of genomic IMERV sequences and IMERV activity, as reported here for 129/Sv mice, might therefore be associated with immunological aberrations. Indeed, mice of the 129 strain show deficits in macrophage recruitment as well as NK and B cell activation [29]–[31]. The causes of these deficiencies have remained elusive, however, the macrophage impairment was reported to be a polygenic trait [29], which might be consistent with the absence of IMERV-related genes.

Regarding the putative immunological impact of IMERV, control of IMERV-like activity may be important, all the more since retroviral activity in general increases the risks of insertional modifications of the genome or the emergence of new retroviral pathogens [32]. The PrP function suggested here might well extend to the control of further retroviral elements in addition to IMERV, as indicated by the potential of PrP revealed in *in vitro* studies [20], [21], [33]. Still, for individual animals the restriction of retroviral activity by PrP might be dispensable, like any proposed role of PrP, as implicated for instance by the viability of inbred PrP-deficient laboratory mice. However, it might be vital at the species level to limit risks posed by the activity of endogenous retroviral elements.

In summary, our experiments have revealed, by correlation, a crucial role of PrP expression in the limitation of a common, but previously unrecognized, phenomenon in the mouse spleen, namely the massive activity of endogenous retroviruses in germinal centers after various immune-stimulations. The biological significance of transient retroviral activity in the spleen and anti-retroviral PrP function remains to be established. Yet, our findings offer an intriguing new viewpoint from which the conceptual conflicts thus far associated with PrP function and TSE pathophysiology may be resolved.

## Materials and Methods

### Mice

All mice were bred and maintained under SPF-conditions and experiments performed in accordance with institutional and Swiss-national guidelines. Wild-type C57BL/6 and 129/Sv mice were purchased from the Institute of Laboratory Animals (Vetsuisse Faculty, University of Zürich, Switzerland). DBA/2 and BALB/c mice were obtained from Harlan Netherlands (Horst, Netherlands). *Prnp^o/o^* (Zürich line, mixed C57BL/6x129/Sv background) and *Prnp^−/−^* (Nagasaki line, C57BL/6) mice were kindly provided by Adriano Aguzzi (Institute of Neuropathology, University of Zürich, Switzerland).

### Virus infection of mice

VSV-Indiana (VSV-IND) growth and infection were performed as described before [16]. LCMV strain WE was originally obtained from Fritz Lehmann-Grube (Heinrich Pette Institute, University of Hamburg, Germany) and propagated on L929 and/or MC57 cells. Mice were infected with 2×10^2^ plaque forming units (PFU) LCMV-WE injected intravenously as indicated. To avoid immune-pathological destruction of splenic microarchitecture, mice were depleted of CD8+ T cells prior to LCMV infection by monoclonal antibody (YTS 169.4) as previously described.

### Non-viral immune-stimulation

Mice were immunized intravenously with IC, as previously described [16], or with LPS (15 µg intravenously/mouse; DIFCO Laboratories, Detroit, USA). 1.5ml thioglycolate 3% (DIFCO 0256-17) was administered intraperitoneally. Because VSV infection was the most efficient immune-stimulus for C57Bl/6 mice, it was chosen for comparative experiments comprising other mouse strains. Successful immune-stimulation was associated with significantly increased spleen net weight, in comparison to naïve controls of the same experimental series, and was readily achieved with C57Bl/6, DBA/2, 129/Sv and the Prnp knockout mice. However, BALB/c mice exhibited increased spleen weight in only one out of two experimental series. BALB/c data of the successful series are shown.

### Electron microscopy

From mouse spleens that were perfusion-fixed with 4% formaldehyde and 0.1% glutaraldehyde small pieces of white pulp were dissected. For plastic embedding, white pulp samples were post-fixed in 1% glutaraldehyde over-night and subjected to standard procedures of osmification, dehydration and Epon embedding. Ultrathin plastic sections were picked up on copper grids and contrasted with uranyl acetate and lead citrate. For immunogold-TEM white pulp samples were immersed for 48 h in 2 M sucrose containing 15% polyvinyl pyrrolidone (10 kDa; Sigma), mounted on aluminum pins and frozen and stored in liquid nitrogen. Ultrathin cryosections were prepared according to Tokuyasu [34] and picked up on nickel grids. Incubation of sections was performed by floating the grids on droplets of the respective solution. Immunolabelling was carried out with specific antibodies diluted in conditioning buffer (PBS containing 0.5% milk powder and 0.02% Tween). PrP-specific immunostaining was achieved with rabbit serum XN [16] at a dilution of 1∶200. MLV Env-specific immunostaining employed rat monoclonal antibody 83A25 [35] at 1∶2 and subsequent rabbit-anti rat (Jackson ImmunoResearch Laboratories Inc., West Grove, PA) at 1∶300. Antibody binding was indirectly gold-labeled by incubation with 8- and 12-nm gold-complexed protein A, respectively. Immunolabelled sections were embedded and stained with methylcellulose and uranyl acetate according to Tokuyasu [34]. Micrographs were taken on a Zeiss EM 910.

### Immunohistochemistry

Semithin sections of 400 nm were prepared from sucrose-infiltrated aldehyde-fixed spleen tissue as for ultrathin cryotomy. Preparation of cryostat sections from non-fixed spleen samples and immunostaining were carried out as previously described [16]. Primary antibodies were the following: PrP-specific rabbit antiserum XN at 1∶600, MLV Env-specific rat antibody 83A25 at 1∶3, MLV gp71-specific goat serum (kindly provided by Roland Friedrich, Microbiology and Virology, University of Giessen, Germany) at 1∶3000, MLV p30-specific goat serum (kindly provided by Hans Lutz, Vetsuisse Faculty, University of Zürich, Switzerland) at 1∶1500, and Dpl-specific affinity-purified goat antibody Dpl (G-20) (Santa Cruz Biotechnology Inc., Santa Cruz, CA) at 1∶50.

### Western blot and PERT analysis

For Western blot analysis, spleen tissue and cell culture sample preparations, electrophoresis and immunoblot detection were performed as decribed previously [16]. Employed primary antibodies were PrP-specific polyclonal rabbit antibody 1B3 [16] at 1∶5000, and MLV p30-specific antibody at 1∶10'000. For determination of splenic reverse transcriptase activity, 10% homogenates of spleen samples prepared for Western blot analysis were diluted 1∶1000 and subjected to PERT assays as described elsewhere [36] (making use of a modification for real-time detection).

### Cell culture experiments

Mouse fibroblasts of the 3T3-Swiss albino cell line were cultured as described previously [16]. For infection with Moloney type MLV culture medium was complemented 1∶2 with 0.45μm-filtered virus-containing supernatant with a titer >10^7^ pfu/ml (viral stock kindly provided by Tatiana Afanasieva, Institute of Neuropathology, University of Zürich, Switzerland) and 8 µg/ml polybrene (Sigma). Medium was exchanged the next morning. Mock-infected and MLV-infected fibroblast cultures were passaged after 3 days and harvested on day 5 for Western blot analysis.

### Specific MLV particle isolation

An approximate amount of 4×10^7^ paramagnetic beads (Dynabeads M450) with covalently linked sheep anti-rat IgG (Dynal Biotech, Oslo, Norway) was washed, resuspended in 800 µl PBS/0.1%BSA and added to 1 ml of MLV Env-specific supernatant 83A25 for incubation over-night at 4°C to obtain MLV-specific beads. A 20% homogenate of immune-stimulated mouse spleen tissue was prepared in 10 mMTris-HCl pH 7.5 supplemented with 1 mM EDTA, 100 mM NaCl and 5% sucrose. The homogenate was centrifuged for 30 min at 10'000g and 4°C, and 0.5 ml of the supernatant was added to washed MLV-specific beads (or, as a negativ control, to an equal amount of original rat IgG-specific beads) and incubated for 2 h at ambient temperature. After incubation beads were collected, washed and processed either for Epon embedding, ultrathin sectioning (100 nm) and EM examination, or for RNA isolation.

### RNA and DNA extraction

Total RNA from paramagnetic bead fractions, spleens, or cells was obtained by direct lysis of the samples in TRI reagent (MRC Inc.) and further processing according to the manufacturers protocol. Putative traces of contaminating DNA were removed with DNA-*free*™ Kit (Ambion Inc.).

DNA was extracted from mouse tail, spleen or brain by decomposing tissue in tail lysis solution over-night at 55°C. After brief centrifugation of the lysate, DNA was precipitated from the supernatant with isopropanol. Precipitate was washed with 70% ethanol, air-dried and reconstituted in 50 mM Tris-HCl pH 8.

### Reverse transcription and PCR amplification

Reverse transcription on RNA extracted from paramagnetic bead fractions was performed using the SuperScript™ First-strand Synthesis System for RT-PCR (Invitrogen) with random hexameric primers according to the manufacturers instructions. Subsequent PCR amplification employed the following primers: forward 5′-ataacccagggacctaatgagtc-3′ for broad Friend-MLV genome-related specificity, forward 5′-ataacacaagggcccaatga-3′ for broad Moloney-MLV genome-related specificity, and reverse 5′-cttccacccgcttgttga-3′ for both of them.

### Cloning and sequencing

PCR products were isolated and purified with the QIAquick Gel Extraction Kit (Qiagen). Cloning was performed by use of the pGEM®-T Easy Vector System kit (Promega) and competent *E. coli* JM109 cells (Promega). Plasmids were purified with the Plasmid Midi Kit (Qiagen) and the inserts sequenced on an ABI 377 sequencer using Big Dye terminator chemistry (Applied Biosystems) and custom primers.

### RNA and DNA quantification by real-time PCR

Real-time RT-PCR analysis of RNA was performed as described previously for GAPDH (internal standard) and PrP-related RNA [16]. The primer-probe set with broad specificty for MLV was: forward 5′-aagcgggtggaagacatcc-3′, reverse 5′-agcccgctcaagaggttgt-3′, probe FAM-5′-ccccaccgtgcccaaccct-3′-TAMRA. The primer-probe set specific for IMERV-1 was: forward 5′-cactttgagggatcaggagcc-3′, reverse 5′-cttctaggtttagggtcaacacctgt-3′, probe FAM-5′-aggttgtgggaccaaaaggacagcc-3′-TAMRA. For IMERV-2-related RNA, the set was: forward 5′-cacttcgagggatcgggagct-3′, reverse 5′-cctctatgccaagggtcaacacctgc-3′, same probe as for IMERV-1.

The procedure for real-time PCR analysis of DNA was identical to the one for RNA analysis except that the template nucleic acid was 5 ng DNA per reaction and the reverse transcription step was omitted.

## Supporting Information

Figure S1 TEM of ultrathin cryosections through the germinal center of a mouse spleen showing an FDC (F) with its labyrinthine extensions between lymphocytes (L). The wild-type mouse of the C57Bl/6 strain was immune-stimulated 8 days before by intravenous IC. PrP-specific immunogold labelling (small black dots) is restricted to the FDC extensions. Retrovirus-like particles can also be discerned, with the morphology of mature (arrows) and immature budding virus (arrowhead). Bar, 500 nm.
(1.40 MB TIF)

Figure S2 Immunofluorescence staining obtained with antibodies directed against MLV Env, gp71 and p30 on consecutive cryosections of spleens of C57Bl/6 mice on day 8 after VSV infection. On each section double-staining revealed the MLV protein in conjunction with PrP. Bar, 100 μm.
(0.09 MB PDF)

Figure S3 Correspondence of mature MLV p30 abundance and reverse transcriptase (RT) activity in mouse spleens. Homogenates from naïve (first from the left) and immune-stimulated C57BL/6 spleens were analysed by Western blot for the p30 protein band at the 30 kDa position (immunoblots at bottom) and by PERT assay for RT activity.
(0.05 MB PDF)

Figure S4 Isolation and amplification of immune-activated MLV-related RNA sequences. (A) Immunoabsorptive isolation of viral particles. Paramagnetic beads were incubated in homogenate of immune-stimulated C57BL/6 spleens, washed and then processed for RNA isolation or for visual control by TEM. Electron micrographs of ultrathin plastic sections show beads without specificity for MLV (left panel), and the surface of an Env-specific bead with bound viral particles (right panel). Bar, 1 μm. (B) MLV-specific RT-PCR products obtained with RNA preparations from immunoabsorptive bead samples. Reverse transcription and amplification of RNA from cultivated Moloney MLV (positive control, lane 1), from non-specific bead samples (lanes 2 and 4) and from Env-specific bead samples (lanes 3 and 5) were performed with primer pairs specific for Moloney type (lanes 1–3) and for Friend type MLV sequence (lanes 4 and 5), respectively. Visible bands (lanes 1 and 5) represent products of the expected size of 1.3 kilobases. Marker, lane 6.
(0.14 MB PDF)

Figure S5 Increased PrP and p30 expression by 3T3 fibroblasts following infection with Moloney MLV. Retrovirus infection was established within 5 days with cell passage in fresh medium. Per experimental group, i.e. control cells (− MLV) and infected cells (+MLV), lysates of three cell cultures were pooled for analysis in the presented Western blots, in order to obtain averaged results.
(0.03 MB PDF)

Figure S6 Dpl and PrP expression in splenic FDC networks of Prnp−/− (Nagasaki), C57Bl/6 wild-type Prnp+/+ mouse and F1 offspring of Prnp−/− (Nagasaki)/C57Bl/6 wild-type crossing (Prnp+/−), on day 8 after VSV infection. Dpl and PrP were revealed by immunofluorescence double-staining. Bar, 100 μm.
(0.09 MB PDF)
