# Supplementary figures and images for: Induced Prion Protein Controls Immune-Activated Retroviruses in the Mouse Spleen
Source: PLoS One. 2007 Nov 7;2(11):e1158. doi: 10.1371/journal.pone.0001158 (PMC2063463; doi:10.1371/journal.pone.0001158)

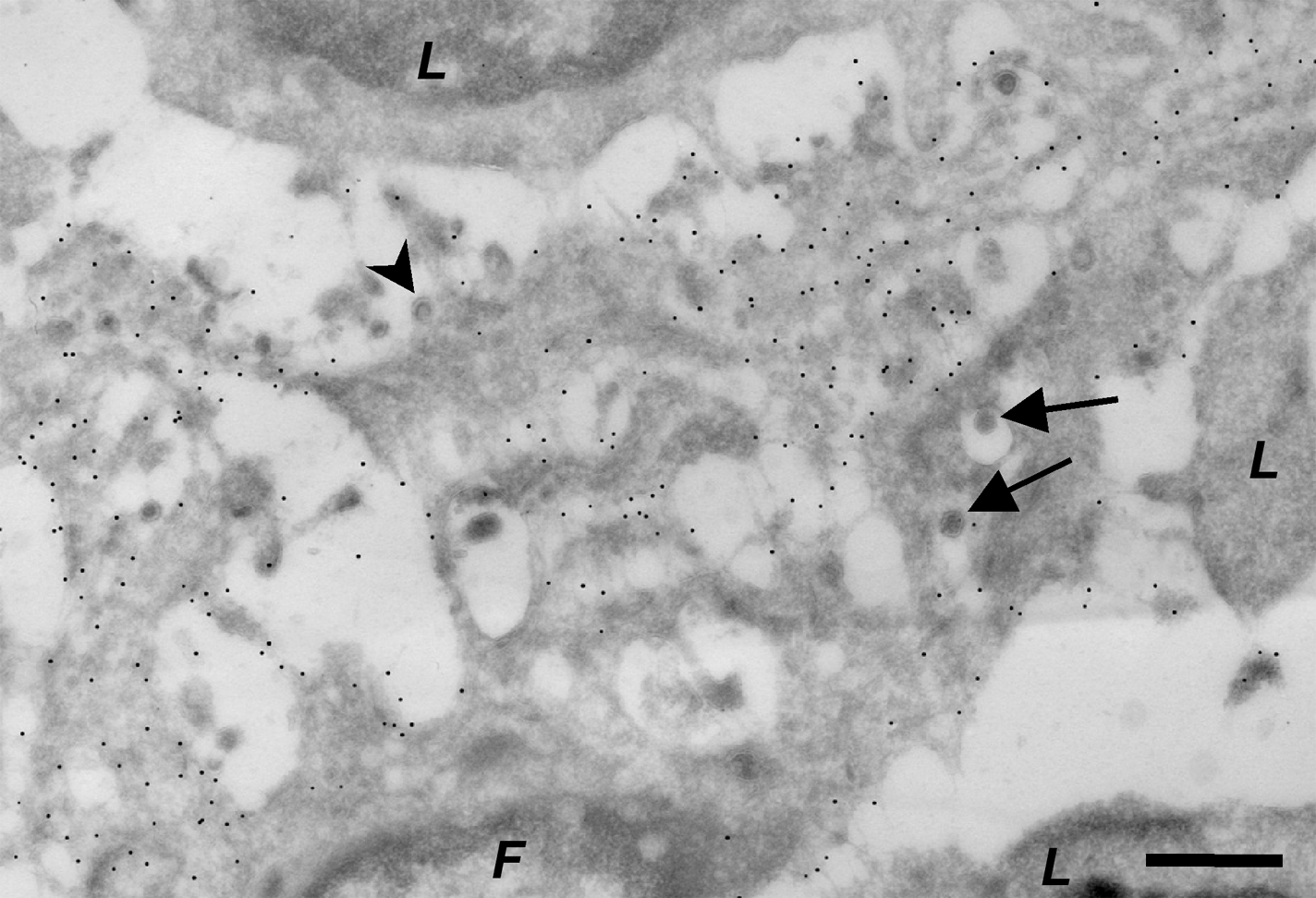

Supplement: Figure S1 — TEM of ultrathin cryosections through the germinal center of a mouse spleen showing an FDC (F) with its labyrinthine extensions between lymphocytes (L). The wild-type mouse of the C57Bl/6 strain was immune-stimulated 8 days before by intravenous IC. PrP-specific immunogold labelling (small black dots) is restricted to the FDC extensions. Retrovirus-like particles can also be discerned, with the morphology of mature (arrows) and immature budding virus (arrowhead). Bar, 500 nm. (1.40 MB TIF) [file pone.0001158.s001.tif]

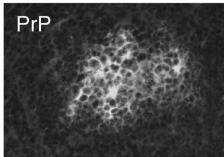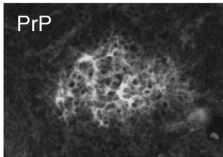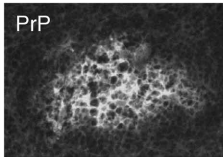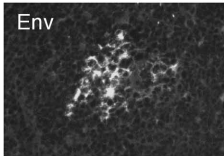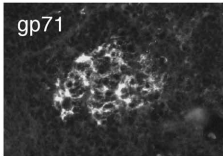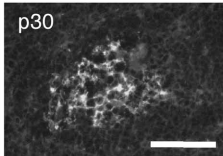

Supplement: Figure S2 — Immunofluorescence staining obtained with antibodies directed against MLV Env, gp71 and p30 on consecutive cryosections of spleens of C57Bl/6 mice on day 8 after VSV infection. On each section double-staining revealed the MLV protein in conjunction with PrP. Bar, 100 μm. (0.09 MB PDF) [file pone.0001158.s002.pdf]

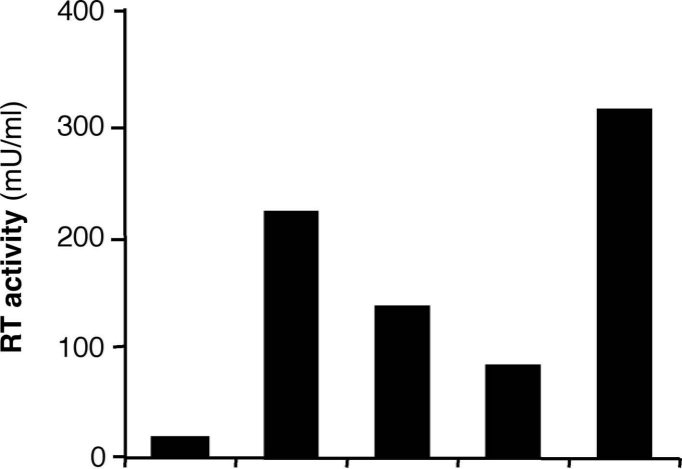

**p30**

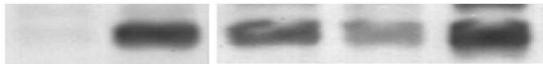

Supplement: Figure S3 — Correspondence of mature MLV p30 abundance and reverse transcriptase (RT) activity in mouse spleens. Homogenates from naïve (first from the left) and immune-stimulated C57BL/6 spleens were analysed by Western blot for the p30 protein band at the 30 kDa position (immunoblots at bottom) and by PERT assay for RT activity. (0.05 MB PDF) [file pone.0001158.s003.pdf]

**A**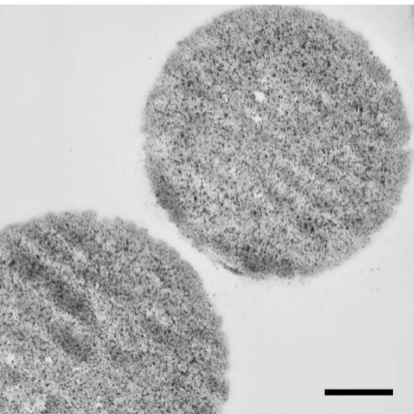

non-specific

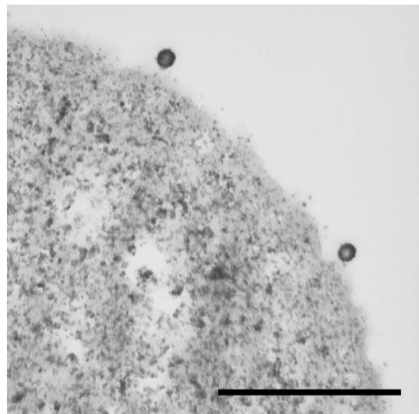

Env-specific

**B**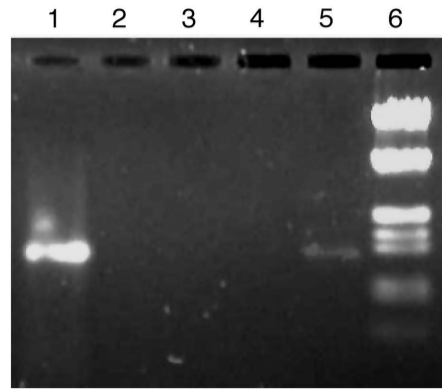

Supplement: Figure S4 — Isolation and amplification of immune-activated MLV-related RNA sequences. (A) Immunoabsorptive isolation of viral particles. Paramagnetic beads were incubated in homogenate of immune-stimulated C57BL/6 spleens, washed and then processed for RNA isolation or for visual control by TEM. Electron micrographs of ultrathin plastic sections show beads without specificity for MLV (left panel), and the surface of an Env-specific bead with bound viral particles (right panel). Bar, 1 μm. (B) MLV-specific RT-PCR products obtained with RNA preparations from immunoabsorptive bead samples. Reverse transcription and amplification of RNA from cultivated Moloney MLV (positive control, lane 1), from non-specific bead samples (lanes 2 and 4) and from Env-specific bead samples (lanes 3 and 5) were performed with primer pairs specific for Moloney type (lanes 1–3) and for Friend type MLV sequence (lanes 4 and 5), respectively. Visible bands (lanes 1 and 5) represent products of the expected size of 1.3 kilobases. Marker, lane 6. (0.14 MB PDF) [file pone.0001158.s004.pdf]

PrP

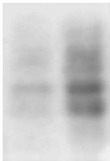

— 29 kD

p30

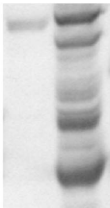

— 70 kD

— 29 kD

Supplement: Figure S5 — Increased PrP and p30 expression by 3T3 fibroblasts following infection with Moloney MLV. Retrovirus infection was established within 5 days with cell passage in fresh medium. Per experimental group, i.e. control cells (− MLV) and infected cells (+MLV), lysates of three cell cultures were pooled for analysis in the presented Western blots, in order to obtain averaged results. (0.03 MB PDF) [file pone.0001158.s005.pdf]

Prnp -/-

F1 +/-

wt +/+

Dpl

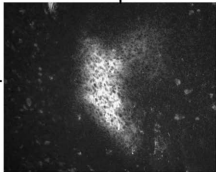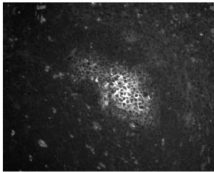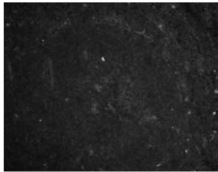

PrP

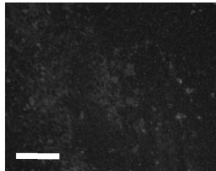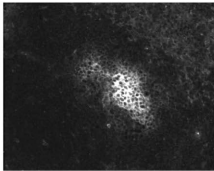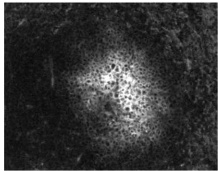

Supplement: Figure S6 — Dpl and PrP expression in splenic FDC networks of Prnp−/− (Nagasaki), C57Bl/6 wild-type Prnp+/+ mouse and F1 offspring of Prnp−/− (Nagasaki)/C57Bl/6 wild-type crossing (Prnp+/−), on day 8 after VSV infection. Dpl and PrP were revealed by immunofluorescence double-staining. Bar, 100 μm. (0.09 MB PDF) [file pone.0001158.s006.pdf]
